# Impact of exhaustive exercise on autonomic nervous system activity: insights from HRV analysis

**DOI:** 10.3389/fphys.2024.1462082

**Published:** 2024-12-03

**Authors:** Weichao Wang, Mingrui Shao, Weiping Du, Yanjun Xu

**Affiliations:** ^1^ School of Physical Education, Northwest Normal University, Lanzhou, Gansu, China; ^2^ School of Physical Education, Shanghai Normal University, Shanghai, China; ^3^ Sports institute, Ningxia Nomal University, Guyuan, Ningxia, China; ^4^ Department of Physical Education, Shanghai University of Finance and Economics, Shanghai, China

**Keywords:** physical exhaustion, heart rate variability, autonomic nervous system, exercise, sympathetic nervous system

## Abstract

**Introduction:**

Exhaustive exercise is a common training method in sports, but its impact on the autonomic nervous system of the human body remains unclear. Understanding the effects of exhaustive exercise on the body and its connection with the autonomic nervous system and central nervous system is crucial for guiding healthy training methods.

**Methods:**

Twenty-three participants were selected, and exhaustive exercise intervention was performed using the Bruce Protocol. By measuring heart rate variability (HRV), the effects of exhaustive exercise on the autonomic nervous system function were analyzed.

**Results:**

After exhaustive exercise, time-domain indices SDNN, RMSSD, and PNN50 all significantly decreased, with changes reaching significant levels (*p* < 0.01). Among them, the decrease in pNN50 was particularly pronounced, with a change rate of −94.55%. Frequency-domain indices VLF, LF, and HF also showed significant decreases (*p* < 0.01), but the ratio of LF to HF showed an upward trend (*p* < 0.01), with LF showing a greater decrease. Nonlinear indices SD1 and SD2 showed extremely significant decreases (*p* < 0.01), and the SD2/SD1 ratio showed a significant increase (*p* < 0.01), indicating significant changes in HRV nonlinear characteristics after exercise.

**Discussion:**

Exhaustive exercise leads to a decrease in autonomic nervous system activity and an increase in sympathetic nervous system activity. These findings underscore the profound impact of exhaustive exercise on the autonomic nervous system, with implications for understanding the physiological responses to intense physical exertion. Further research is warranted to explore the long-term effects of exhaustive exercise on autonomic regulation and its potential implications for training methodologies and athlete health.

## 1 Introduction

Exhaustive exercise, characterized by pushing the body to its physiological and psychological limits, is a central component of athletic training programs worldwide (Johnson, 2009; Liao et al., 2020). Athletes in various disciplines, from endurance runners and cyclists to powerlifters, rely on such intense training regimens to develop strength, endurance, and resilience (Adami et al., 2022; Maestroni et al., 2020). This demanding approach has proven critical for building peak physical capabilities and achieving competitive success (Ammar et al., 2018a; Fokkema et al., 2020). Beyond the benefits of enhanced performance, exhaustive exercise triggers a cascade of physiological responses that, if better understood, could enhance not only athletic potential but also the safety and effectiveness of training methods.

During exhaustive exercise, one of the most significant physiological responses is the alteration in heart rate variability (HRV), a key marker of autonomic nervous system (ANS) activity (Michael et al., 2017). The ANS, composed of the sympathetic and parasympathetic branches, regulates essential functions like heart rate, blood pressure, and respiration, all of which are heavily impacted by intense physical exertion (Makivić et al., 2013; Thomas et al., 2019). In strenuous exercise, the sympathetic branch dominates, triggering the “fight or flight” response, while the parasympathetic branch aids recovery post-exercise (Wehrwein et al., 2016; Wisneski, 2017). Decreased HRV observed after maximal exertion indicates heightened sympathetic activity and reduced parasympathetic influence, reflecting the body’s stress response and need for recovery (Makivić et al., 2013). These shifts in HRV are essential for understanding the body’s resilience and recovery, offering insights into how individuals adapt to physical stress. However, further research is needed to precisely capture these dynamics, particularly through longitudinal studies and advanced physiological monitoring, which can elucidate the intricate balance between exhaustive exercise and autonomic regulation.

While the physiological responses to exercise, particularly in relation to heart rate variability (HRV) and autonomic nervous system (ANS) regulation, are well-documented, significant gaps remain in understanding the specific mechanisms and implications of these changes during and after exhaustive physical activity (Kenney et al., 2022; Maud and Foster, 2006). Much of the existing research has focused on moderate to sub-maximal exercise intensities, with fewer studies examining the effects of exhaustive, near-maximal exertion on HRV. Additionally, most studies have centered on resting HRV as a general health and recovery marker, without thoroughly investigating HRV fluctuations during extreme exercise and the immediate recovery period (Kenney et al., 2022). Moreover, limited attention has been given to how specific HRV components, such as low-frequency and high-frequency power, respond under exhaustive conditions, and how these changes might inform training and recovery strategies. Addressing these gaps requires a more comprehensive investigation into how exhaustive exercise impacts autonomic balance and physiological recovery.

The primary objective of this study is to analyze the effects of exhaustive exercise on the ANS, with a specific focus on how it influences HRV regulation. By elucidating these effects, this research aims to inform evidence-based strategies for optimizing athletic performance and minimizing the risks of overtraining and injury. Understanding how exhaustive exercise modulates autonomic function can empower coaches, athletes, and healthcare professionals to tailor training regimens effectively, maximizing performance while ensuring athlete wellbeing.

## 2 Methods

### 2.1 Participants

Thirty male college students, aged 18 to 19, from Shaanxi Normal University participated in a preliminary investigation. Participants were excluded having a history of cardiovascular diseases (heart disease, coronary artery disease, arrhythmias) to reduce confounding effects on HRV data, or severe respiratory conditions (asthma, chronic obstructive pulmonary disease) due to their impact on respiratory rate and HRV outcomes. Additionally, individuals with serious psychiatric disorders (depression, anxiety, bipolar disorder) were excluded to control for potential influences on autonomic nervous system stability. Those taking medications affecting heart rate or HRV (beta-blockers, antidepressants, sedatives), regular smokers, heavy alcohol users, individuals with hypertension, diabetes, or other metabolic disorders, and those who had engaged in vigorous physical activity within the previous week were also excluded to ensure reliable HRV and blood pressure measurements.

To ensure that the participants were capable of engaging in high-intensity exercise, they underwent a physical fitness assessment, which included 100-m and 800-m runs, both required to meet the national third-level standards. During the testing process, two individuals withdrew, and four did not pass the 800-m run. Consequently, 24 individuals were included as the formal research subjects. All participants were right-handed and had no history of neurological disorders such as epilepsy or brain trauma (Chapman and Chapman, 1987). Informed consent was obtained from all subjects prior to participation, and the study received ethical approval from the Ethics Committee of Shaanxi Normal University. The basic characteristics of participants are detailed in Table 1.

**TABLE 1:** Basic information on participants.

Age (years)	18.76 ± 0.87
Height (cm)	182 ± 4.78
Body weight (kg)	79 ± 8.88
Basal heart rate (Bit/min)	59.23 ± 7.55
BMI (kg/m^2^)	24.33 ± 3.29
Skeletal muscle (%)	39.48 ± 3.72
Body fat percentage (%)	13.92 ± 4.63
Basal metabolism (Kcal)	1883 ± 187.2
100 m (s)	≤12.4
800 m (s)	≤136

### 2.2 Establishment of exhaustive exercise mode

The exhaustive exercise model was conducted at the Equi-Speed Strength Laboratory, Shaanxi Normal University. Participants were instructed to abstain from alcohol, late nights, and medication for 1 week prior to the experiment, and to avoid smoking, coffee, or strong tea within 72 h before the experiment to ensure optimal physical and mental states. The experimental intervention utilized the Bruce Protocol, a progressive exercise model on a motorized treadmill (h/p/cosmos cos10253, Germany), designed to induce exhaustion. The Bruce Protocol consists of 7 stages, each with predetermined speed and incline, progressively increasing in intensity. Due to its significant level increments, the Bruce Protocol effectively elicits distinct physiological responses, making it a common choice in cardiopulmonary exercise experiments involving physically fit adolescents.

The exercise session occurred between 9:00 a.m. and 12:00 p.m. for all participants. Each participant followed the same protocol, with the treadmill advancing to the next stage every 3 minutes. Participants wore a portable blood pressure monitoring device (Omron, Liaoning, China) throughout the exercise session to continuously record dynamic blood pressure changes during the exercise. Participants’ subjective feelings were assessed at the end of each stage using the Rating of Perceived Exertion (RPE) scale. The exercise session was terminated based on established criteria (Zhao et al., 2024) if any three of the following conditions were met:

1. Behavioral indicators: Breathing difficulty accompanied by profuse sweating;
2. Heart rate: Approaching or reaching maximum heart rate (220 - age);
3. Blood pressure changes: Systolic blood pressure >150 mmHg or diastolic blood pressure >75 mmHg;
4. RPE level: Reaching an RPE value of 18–19, indicating exhaustion despite encouragement.

All participants completed the Bruce Protocol with the same exercise duration and intensity levels. The treadmill advanced through stages every 3 minutes, ensuring uniformity in the exercise duration and intensity across all subjects. The total duration of exercise varied depending on the participant’s fitness level, but all were assessed within the protocol’s standardized seven stages.

### 2.3 HRV data acquisition and processing

The GTX9 (United States) monitor, along with ActiLife and Kubios software, was used to record HRV time-domain, frequency-domain, and nonlinear indices. Upon launching the ActiLife software and connecting the GT9-X device, participants’ basic information (ID, gender, age, weight) was entered. The R-R interval data collection was set from 8:00 a.m. to 12:00 p.m. at a sampling rate of 30 Hz. Participants wore both the GT9-X and a Polar watch throughout the experiment. After the experiment, R-R interval values during the first 5 min of quiet sitting before exercise and during the fifth to 10th minute of quiet sitting post-exercise were downloaded using ActiLife software. These values were analyzed using Kubios software to compute HRV indices.

For time-domain indices, R-R interval standard deviation (SDNN), root mean square of successive R-R interval differences (RMSSD), and the percentage of successive R-R intervals differing by more than 50 ms (pNN50) were selected. Frequency-domain indices included the mean output power of very low frequency (VLF), low frequency (LF), high frequency (HF), and the ratio of LF to HF. Nonlinear indices comprised short axis (SD1), long axis (SD2), and the ratio of SD2 to SD1 in Poincaré plots.

### 2.4 Physiological load index

In this study, the physiological load index was calculated to assess the relative cardiovascular strain during exercise. The index was determined using the following formula:

$$Physiological\;Load\;Index=\frac{{HR}_{peak}+{HR}_{rest}}{2\times {HR}_{rest}}$$

where HR_peak_ represents the maximum heart rate achieved during the exercise session, and HR_rest_ denotes the baseline resting heart rate measured prior to exercise. This index was used to quantify the cardiovascular load relative to the individual’s resting condition, providing an objective measure of exercise intensity and its physiological impact.

### 2.5 Statistical analysis

The normality of the data was assessed using the Shapiro-Wilk (S-W) test, while homogeneity of variance was evaluated using the Levene test. The HRV data did not follow a normal distribution, thus non-parametric tests were employed. The Wilcoxon signed-rank test assessed differences in HRV indices before and after exhaustive exercise. Descriptive statistics were presented as median and quartiles (M [P25∼P75]). All analyses were two-tailed, with significance levels set at *p* < 0.05 for significant differences and *p* < 0.01 for highly significant differences.

## 3 Results

### 3.1 Exhaustion model evaluation results

All participants exhibited pronounced symptoms of dyspnea and profuse sweating, reflecting the high physiological load reached during the intense physical activity. Heart rate approached their maximal heart rate, with an average value of 199 ± 12 beats per minute, close to the theoretical maximum heart rate (220 minus age), indicating the high intensity of the exercise. Additionally, the subjective perception of fatigue was evident, with a RPE score of 19 ± 4, reflecting extreme fatigue and discomfort experienced by the participants. In terms of blood pressure, the systolic blood pressure exceeded 150 mmHg in all participants, with a mean value of 155.47 ± 17.56 mmHg, while the diastolic blood pressure was also higher than 75 mmHg, averaging 75.95 ± 13.71 mmHg. These four physiological indicators align with the established criteria for exhaustive exercise in this experiment, further validating the exercise protocol and confirming the adequacy of the exercise intensity.

**FIGURE 1:**
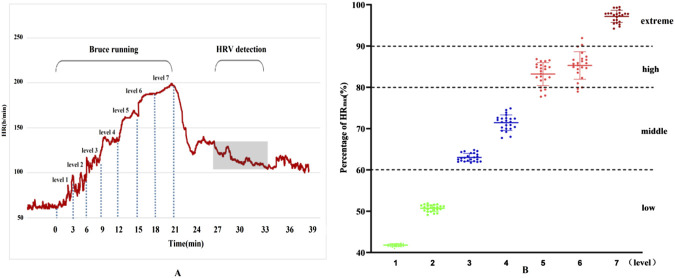
Results of heart rate changes and exercise load during exercise. **(A)** Dynamic heart rate curve throughout exercise; **(B)** Heart rate percentage.

Using the Percentage of Maximum Heart Rate (HRmax), the intensity of exercise was categorized as follows: very High-Intensity Exercise: Exceeding 90% HRmax (about 85% VO₂max); high-Intensity Exercise: 80%–90% HRmax (75%–84% VO₂max); moderate-Intensity Exercise: 60%–80% HRmax (50%–74% VO₂max); low-Intensity Exercise: Below 60% HRmax. The seven stages of the Bruce program, shown in Figure 1, exhibit different levels of intensity. During stages one and two, the participants experienced low-intensity exercise. As the intensity increased, stages three and four reached moderate levels, stages five and six fell into high-intensity, and by stage seven, exercise intensity was extremely high. The Physiological Load Index also supported these findings, indicating a shift from moderate to high load from stage 4 onwards.

**FIGURE 2:**
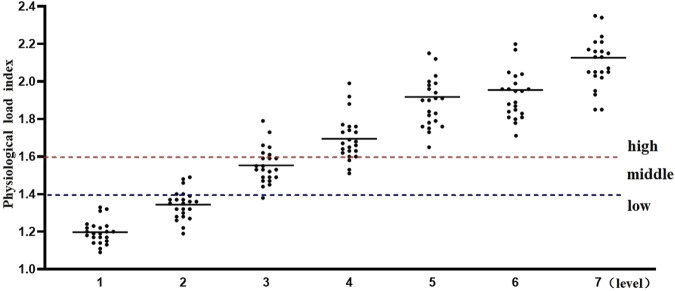
Physiological load index Physiological load index = (HR peak + HR rest)/2/HR rest.

Additionally, the physiological load index is commonly used to assess the intensity of physiological load during exercise training sessions (Reganova et al., 2023). When the physiological load index falls within the range of 1.4–1.6, the physiological load is considered moderate. Values above or below this range indicate either excessive or insufficient physiological load. As shown in Figure 2, the results of this study indicate that, for physical education students, the first and second stages of the Bruce Protocol correspond to low physiological load, while the third stage represents a moderate physiological load. From the fourth stage onward, the physiological load enters the high-exercise intensity range, and by the seventh stage, it exceeds the threshold for high-exercise physiological load.

### 3.2 HRV results before and after exhaustive exercise

The Welcoxon-test was conducted to analyze the changes in time-domain, frequency-domain, and nonlinear indices before and after exhaustive exercise. The characteristics of HRV time-domain indices before and after exhaustive exercise are presented in Table 2. Comparing with pre-exercise, the SDNN, RMSSD, and PNN50 exhibited highly significant decreases after exhaustive exercise, with Z values of −4.541, −4.517, and −4.514, respectively (*p* < 0.01). However, the decrease in pNN50 seemed more pronounced, with a change rate of −94.55%, suggesting a potential relationship with vagal nerve suppression induced by exhaustive exercise.

**FIGURE 3:**
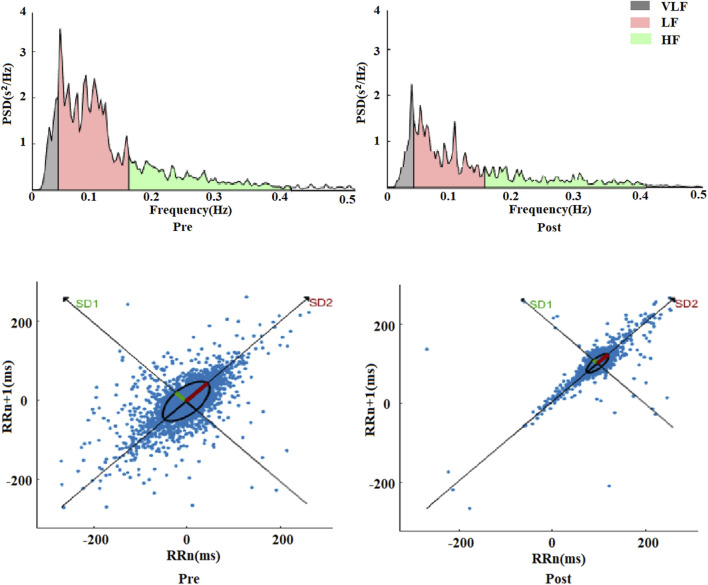
Schematic diagram of variation of nonlinear index Poincare scatter plot during motion.

Similarly, comparing with pre-exercise, the HRV frequency-domain indices VLF, LF, and HF displayed significant decreases after exhaustive exercise, with Z values of −3.556, −4.252, and −4.252, respectively (*p* < 0.01). However, the ratio of LF to HF showed a gradient increase trend, with a Z value of −3.949 (*p* < 0.01). Comparing LF and HF, the decrease in HF was more substantial, which was the primary reason for the increase in LF/HF ratio. Besides statistical analysis of frequency-domain values before and after exercise, fast Fourier transformation was applied to R-R intervals to obtain power spectral density versus frequency plots, as shown in Figure 3. The frequency-domain indices of HRV before and after exhaustive exercise reflected in the power spectral plots exhibited consistent trends.

**TABLE 2:** Comparison of HRV time domain indicators before and after exhaustive exercise M (P25∼P75).

Abbreviation	ANS branch activated	ANS branch activated	Pre-exercise	Post-exercise	Z	p	Hedge’s g	CIs 95%
SDNN (ms)	Standard Deviation of NN Intervals	Parasympathetic	40.43 (29.54∼47.34)	8.70 (6.57∼11.71)	−4.541	0.000	9.70	6.56, 12.84
RMSSD (ms)	Root Mean Square of Successive Differences	Parasympathetic	39.05 (25.14∼49.50)	6.19 (4.48∼8.51)	−4.517	0.001	8.74	5.52, 11.95
PNN50 (%)	Proportion of NN50 Intervals	Parasympathetic	20.17 (3.94∼28.71)	1.10 (0.02∼1.99)	−4.514	0.000	2.91	1.21, 3.44
VLF (ms2)	Very Low-Frequency Power	Sympathetic	62.87 (48.08∼86.26)	19.11 (15.96∼33.20)	−3.556	0.000	2.32	1.21, 3.44
LF (ms2)	Low-Frequency Power	Sympathetic	620.50 (314.33∼1,163.60)	135.46 (118.66∼179.05)	−4.252	0.000	1.39	0.62, 2.17
HF (ms2)	High-Frequency Power	Parasympathetic	494.18 (186.78∼809.18)	107.59 (104.34∼113.56)	−4.252	0.000	1.72	0.87, 2.58
LF/HF	Low-Frequency to High-Frequency Ratio	Sympathetic and Parasympathetic	1.68 (0.61∼3.59)	4.83 (3.31∼6.36)	−3.949	0.002	2.22	1.12, 3.33
SD1 (ms)	Short-Term SD of Poincare Plot	Parasympathetic	26.86 (17.80∼35.06)	3.38 (3.17∼6.02)	−4.517	0.000	3.60	2.39, 5.01
SD2 (ms)	Long-Term SD of Poincare Plot	Sympathetic	45.62 (35.88∼58.74)	10.62 (8.35∼15.92)	−4.541	0.000	3.70	2.39, 5.01
SD2/SD1	Ratio of Long-Term to Short-Term SD	Sympathetic	1.93 (1.67∼2.25)	2.59 (2.21∼3.32)	−3.592	0.000	2.39	1.42, 3.36

The frequency range of 00.4 Hz corresponded to VLF, 0.040.15 Hz represented LF, and 0.150.4 Hz reflected HF changes. From the plots, it was observed that the power spectra exhibited a decreasing trend within the 0.5 Hz range after exhaustive exercise compared to pre-exercise. These results suggest that the effects of exhaustive exercise on HRV frequency-domain indices align with those observed in the power spectral plots.

Comparing with pre-exercise resting states, the post-exercise Poincaré scatter plot short axis SD1 and long axis SD2 displayed highly significant decreases, with Z values of −4.517 and −4.541, respectively (*p* < 0.01). However, the SD2/SD1 ratio showed a highly significant increase, with a Z value of −3.592 (*p* < 0.01). Regarding nonlinear indices, it appeared that the change rate of short axis SD1 was seemingly more pronounced than that of long axis SD2. Figure 3 illustrates a visual representation of Poincaré scatter plots in HRV nonlinear analysis. Before exercise, the scatter points were relatively dispersed and elliptically shaped. However, after exhaustive exercise, the scatter plot exhibited a significant transformation, with the ellipse narrowing and shrinking in area, accompanied by a notable trend of point concentration.

## 4 Discussion

The primary objective of this study was to investigate the physiological and autonomic nervous system responses to exhaustive exercise in physically fit college students, using HRV analysis to assess changes in autonomic regulation. This investigation aimed to provide insight into the impact of extreme physical exertion on autonomic control, particularly examining shifts in sympathetic and parasympathetic balance.

The results indicate that exhaustive exercise induces substantial physiological responses, as evidenced by elevated heart rates, systolic and diastolic blood pressure, and perceived exertion levels. In alignment with previous literature, these physiological responses fulfilled established exhaustion criteria, supporting the intensity levels achieved during the exercise protocol. In addition, HRV indices showed significant reductions in both time-domain (SDNN, RMSSD, pNN50) and frequency-domain (VLF, LF, HF) parameters, with a concurrent increase in the LF/HF ratio. The observed decrease in HF, a parasympathetic marker, suggested a shift towards sympathetic dominance. Nonlinear HRV indices, particularly the SD1 and SD2 components of the Poincaré plot, further reflected autonomic adaptation to intense exercise, with a narrowing of the scatter plot suggesting reduced variability post-exercise. These findings support the initial hypothesis that exhaustive exercise would lead to marked reductions in HRV indices, signaling a heightened sympathetic response coupled with reduced parasympathetic modulation.

### 4.1 Exhaustion model evaluation results

The physiological markers collected in this study align with recognized exhaustion thresholds in the literature. The Bruce Protocol, commonly employed for evaluating exercise intensity, allowed participants to progress from low-to high-intensity stages, with stage 7 clearly achieving very high exertion levels. The progressive increase in heart rate and blood pressure mirrors previous research findings, which demonstrate a proportional increase in physiological load as exercise intensity advances (Hanson et al., 2016; Will and Walter, 1999). This pattern suggests that the protocol effectively induced exhaustion and provided a reliable model for examining autonomic responses to graded intensity.

### 4.2 Time-domain HRV analysis

The results of this study indicate that after exhaustive exercise, heart rate variability indices SDNN, RMSSD, and pNN50 all significantly decrease, suggesting that exhaustive exercise significantly affects the autonomic regulation ability of the heart. Specifically, the decrease in SDNN reflects the overall reduction in heart rate variability, while the decreases in RMSSD and pNN50 mainly reflect the reduction in parasympathetic nervous system activity (Almeida-Santos et al., 2016; Ishaque et al., 2021). It is worth noting that although these indices all show a downward trend, the decrease in pNN50, which represents parasympathetic nervous system activity, seems to be more significant. This suggests that exhaustive exercise exerts a more significant stimulus on the hearts of physical education college students, particularly in terms of inhibitory effects on the vagus nerve. In terms of time domain indices, clinically, SDNN <50 ms is generally considered to reflect significantly increased sympathetic nervous system activity, while RMSSD <25 ms represents significant inhibition of the vagus nerve (Li et al., 2009). This study found that compared to before exercise, SDNN decreased by 73.06%, RMSSD decreased by 84.15%, and was below 10 ms, while pNN50 decreased by 94.55%. In comparison, previous studies have shown that after high-intensity exercise in high-altitude training, SDNN and RMSSD decreased by only 16.42% and 21.92% (Chang et al., 2013), respectively, indicating that the vagus nerve-mediated arterial pressure reflex sensitivity is greatly affected by exhaustive exercise, leading to a weakened regulatory capacity of the vagus nerve on heart function in physical education college students. Exhaustive exercise significantly increases cardiac disorder compared to general high-intensity exercise (Guiraud et al., 2010).

### 4.3 Frequency-domain HRV analysis

In addition, we performed fast Fourier transform on the R-R interval to obtain the frequency-domain power spectral graph with power spectral density as the ordinate and frequency as the abscissa. Three peaks in the graph are shown: the 0–0.4 Hz region represents VLF, the 0.04–0.15 Hz region represents LF peak, and the 0.15–0.4 Hz reflects the HF variations (Kim et al., 2009). Compared with pre-exercise, the VLF, LF, and HF of HRV frequency domain indices after exhaustive exercise also showed a significant downward trend, indicating a significant weakening of the autonomic nervous system regulation after exhaustive exercise. However, despite the significant decrease in LF and HF, the LF/HF ratio showed a trend of gradient increase. This phenomenon is mainly due to the larger decrease in HF. HF usually reflects parasympathetic nervous activity, and its significant decrease indicates a more pronounced parasympathetic inhibition (Elghozi and Julien, 2007). The increase in LF/HF ratio indicates a relative increase in sympathetic nervous activity, although LF itself is also decreasing, but to a lesser extent than HF (Khan et al., 2019). Traditionally, the LF component of HRV has been associated with ANS regulation, which are thought to be primarily mediated by sympathetic activity (Lombardi, 2002). However, recent literature has pointed out that the LF component may not solely reflect sympathetic activity. Rather, it can also arise from central neural mechanisms, such as brainstem regulation, which influence autonomic control over heart rate (Shaffer and Ginsberg, 2017) The HF component, often linked to parasympathetic activity, is greatly influenced by respiratory rhythms. Together, the LF/HF ratio has been used to assess the overall balance between sympathetic and parasympathetic activity, reflecting the autonomic response to exercise, where both central and peripheral mechanisms contribute to its regulation. Generally, the ratio of LF to HF is between 1.5 and 2.0, however, in this experiment, LF and HF decreased by 78.17% and 78.22% respectively after exhaustive exercise compared with before exercise, and LF/HF showed a gradient increase, rising by 65.21% compared with before exercise, deviating significantly from the range value (Hanss et al., 2005). These results have important implications for exercise physiology and training practice. First, they provide detailed evidence that exhaustive exercise significantly affects the balance of the autonomic nervous system, especially by significantly inhibiting parasympathetic activity and relatively enhancing sympathetic activity. This imbalance may be an adaptive response of the body to cope with high-intensity exercise stress. Understanding the changes in these frequency domain indices can help me better design training and recovery plans. For example, by monitoring changes in VLF, LF, and HF, the fatigue level and recovery status of athletes can be more accurately assessed, avoiding negative effects caused by overtraining (Moldovan et al., 2015). The increase in LF/HF ratio can also be used as an important indicator to judge the enhancement of sympathetic nervous activity and the inhibition of parasympathetic nervous activity, thereby taking corresponding adjustment measures in training (Borresen and Lambert, 2008).

### 4.4 Nonlinear HRV analysis

Compared with pre-exercise, both the short axis SD1 and long axis SD2 in the Poincaré plot after exercise show extremely significant decrease trends. This indicates that after exhaustive exercise, both short-term and long-term variability of the heart significantly decrease. SD1 reflects the instantaneous variability of RR intervals, mainly regulated by parasympathetic nervous activity, while SD2 reflects the long-term variability of heart rate, jointly regulated by sympathetic and parasympathetic nerves (Agorastos et al., 2023; Rahman et al., 2018). The significant decrease of both indicates that after exhaustive exercise, the autonomic regulation ability of the heart significantly decreases, especially with a significant inhibition of parasympathetic nervous activity. However, despite the significant decrease in both SD1 and SD2, the SD2/SD1 ratio shows an extremely significant increase. This phenomenon is mainly because the decrease in SD1 is greater, further indicating a more pronounced inhibition of parasympathetic nervous activity after exhaustive exercise. The increase in the SD2/SD1 ratio indicates a relative enhancement of sympathetic nervous activity, although SD2 itself is also decreasing, but to a lesser extent than SD1. These results have important implications for exercise physiology and training practice. The increase in the SD2/SD1 ratio can also be used as an important indicator to judge the enhancement of sympathetic nervous activity and the inhibition of parasympathetic nervous activity, thereby taking corresponding adjustment measures in training.

Research has found that during exercise, under the domination of the sympathetic nervous system, HR and ventricular contractility begin to increase, with sympathetic nerve stimulation mainly mediated by endogenous adrenaline (Garg et al., 2020). As athletes endure increasing exercise loads, the conduction within the heart (such as atrioventricular conduction) also increases continuously, accompanied by a decrease in HRV (Garg et al., 2020). Additionally, autonomic nervous regulation during exercise may be associated with hemodynamic changes, altering the cardiac load. Due to the redistribution of blood throughout the body during exercise, there is increased blood flow in skeletal muscles (Garg et al., 2020), and the fluid shear stress of blood flow changes with the variation in blood flow during exercise, causing changes in the function of pressure-sensitive nerves, leading to a shift in the balance between the vagus and sympathetic nervous systems towards sympathetic dominance, resulting in increased sympathetic nervous activity and changes in LF and HF indices representing sympathetic excitation and vagal inhibition during exercise (Garg et al., 2020). This experiment, based on exhaustive exercise on the Bruce treadmill, belongs to high-intensity exercise, with significant fluctuations in heart rate during exercise, peaking at approximately 202 beats per minute, and the degree of HRV change is extremely significant, especially in the HRV value representing vagal nerve activity. This indicates that under the stress of exhaustive exercise, the vagal nerves of sports science students are inhibited due to the effects of adrenaline released by the adrenal medulla, as well as factors such as cardiopulmonary baroreceptors and hemodynamics (Daniela et al., 2022). Sympathetic nervous activity is significantly enhanced, the dynamic balance of the autonomic nervous system is gradually disrupted, and serious disruptions in cardiac function begin to occur. Multiple studies have shown that increased sympathetic nervous activity accompanied by inhibition of vagal nerve activity-induced cardiac dysfunction is closely related to exercise-related sudden death (Grise, 2012; Tahara et al., 2005). Therefore, compared to general exercise, the likelihood of exercise-induced sudden death greatly increases with exhaustive exercise. This experiment used time-domain and frequency-domain indices of HRV, combined with the application of nonlinear visualization indices scatter plots, to explore the effects of exhaustive exercise on the autonomic nervous system of undergraduate students majoring in sports science, providing a good background for studying the effects of exhaustive exercise loads on sympathetic and vagal nerves in athletes, and further investigating the role of “exhaustive” exercise factors in autonomic nervous balance, exercise fatigue, and exercise-related sudden death.

Based on our findings, we recommend that practitioners incorporate HRV monitoring into the training regimens of individuals engaging in exhaustive exercise. Monitoring HRV before, during, and after high-intensity sessions enables practitioners to assess each individual’s autonomic response and recovery rate, providing a personalized approach to exercise intensity adjustments. Specifically, reduced HRV after training could signal the need for additional recovery time, while stable HRV may allow for safely increasing training loads. This approach could help minimize the risks of overtraining, autonomic imbalance, and injury associated with sustained high-intensity exercise. For instance, our sample primarily included trained athletes, and the generalizability to other populations remains untested. Future research should aim to expand these findings to various demographics and sports to create more universally applicable guidelines. Additionally, more work is needed to determine precise HRV thresholds for recovery needs and performance optimization, enhancing the reliability of HRV as a training tool across different contexts. The controlled laboratory setting, with treadmill-based exercise, may not fully reflect real-world conditions where variables like temperature, humidity, and uneven terrain could impact physiological outcomes. To validate and expand these findings, conducting field studies that simulate practical exercise scenarios is essential. Finally, our short-term study design limits insights into the long-term effects of high-intensity exercise on HRV and autonomic function. Future studies with extended tracking of participants would help clarify how sustained high-intensity training influences autonomic balance, cardiac health, and long-term adaptation.

## 5 Conclusion

This study provides key insights into how exhaustive exercise impacts the autonomic nervous system, particularly through changes in HRV that signal shifts in sympathetic and parasympathetic activity. Our findings reveal that exhaustive exercise significantly reduces HRV indices associated with autonomic recovery, suggesting that systematic HRV monitoring before and after intense workouts could enable practitioners to adjust training intensity based on each athlete’s recovery capacity. Such adjustments could prevent overtraining, reduce injury risk, and optimize recovery. Specifically, these insights support training programs that balance high exertion with individualized recovery periods, potentially enhancing athletic performance, resilience, and long-term health.

## Data Availability

The raw data supporting the conclusions of this article will be made available by the authors, without undue reservation.

## References

[B1] Adami P. Rocchi J. Melke N. De Vito G. Bernardi M. Macaluso A. (2022). Physiological profile comparison between high intensity functional training, endurance and power athletes. Eur. J. Appl. physiology 122, 531–539. 10.1007/s00421-021-04858-3 34853894

[B2] Agorastos A. Mansueto A. C. Hager T. Pappi E. Gardikioti A. Stiedl O. (2023). Heart rate variability as a Translational dynamic biomarker of altered autonomic function in Health and Psychiatric disease. Biomedicines 11 (6), 1591. 10.3390/biomedicines11061591 37371686 PMC10295200

[B4] Almeida-Santos M. A. Barreto-Filho J. A. Oliveira J. L. M. Reis F. P. da Cunha Oliveira C. C. Sousa A. C. S. (2016). Aging, heart rate variability and patterns of autonomic regulation of the heart. Archives gerontology geriatrics 63, 1–8. 10.1016/j.archger.2015.11.011 26791165

[B6] Ammar A. Riemann B. L. Masmoudi L. Blaumann M. Abdelkarim O. Hökelmann A. (2018a). Kinetic and kinematic patterns during high intensity clean movement: searching for optimal load. J. Sports Sci. 36 (12), 1319–1330. 10.1080/02640414.2017.1376521 28895467

[B7] Borresen J. Lambert M. I. (2008). Autonomic control of heart rate during and after exercise: measurements and implications for monitoring training status. Sports Med. 38, 633–646. 10.2165/00007256-200838080-00002 18620464

[B8] Chang H.-A. Chang C.-C. Chen C.-L. Kuo T. B. Lu R.-B. Huang S.-Y. (2013). Heart rate variability in patients with fully remitted major depressive disorder. Acta Neuropsychiatr. 25 (1), 33–42. 10.1111/j.1601-5215.2012.00658.x 26953072

[B9] Chapman L. J. Chapman J. P. (1987). The measurement of handedness. Brain Cognition 6 (2), 175–183. 10.1016/0278-2626(87)90118-7 3593557

[B10] Daniela M. Catalina L. Ilie O. Paula M. Daniel-Andrei I. Ioana B. (2022). Effects of exercise training on the autonomic nervous system with a focus on anti-inflammatory and antioxidants effects. Antioxidants 11 (2), 350. 10.3390/antiox11020350 35204231 PMC8868289

[B11] Elghozi J. L. Julien C. (2007). Sympathetic control of short‐term heart rate variability and its pharmacological modulation. Fundam. and Clin. Pharmacol. 21 (4), 337–347. 10.1111/j.1472-8206.2007.00502.x 17635171

[B12] Fokkema T. van Damme A. A. Fornerod M. W. de Vos R. J. Bierma‐Zeinstra S. M. van Middelkoop M. (2020). Training for a (half‐) marathon: training volume and longest endurance run related to performance and running injuries. Scand. J. Med. and Sci. sports 30 (9), 1692–1704. 10.1111/sms.13725 32421886 PMC7496388

[B13] Garg V. Verma S. Connelly K. A. Yan A. T. Sikand A. Garg A. (2020). Does empagliflozin modulate the autonomic nervous system among individuals with type 2 diabetes and coronary artery disease? The EMPA-HEART CardioLink-6 Holter analysis. Metab. Open 7, 100039. 10.1016/j.metop.2020.100039 PMC7424781 32812924

[B14] Grise K. N. (2012). The effects of exercise training on indices of cardiovascular autonomic neuropathy in STZ-induced type 1 diabetic rats treated with insulin. The University of Western Ontario. (Canada).

[B15] Guiraud T. Juneau M. Nigam A. Gayda M. Meyer P. Mekary S. (2010). Optimization of high intensity interval exercise in coronary heart disease. Eur. J. Appl. physiology 108, 733–740. 10.1007/s00421-009-1287-z 19915859

[B16] Hanson N. J. Scheadler C. M. Lee T. L. Neuenfeldt N. C. Michael T. J. Miller M. G. (2016). Modality determines VO 2max achieved in self-paced exercise tests: validation with the Bruce protocol. Eur. J. Appl. physiology 116, 1313–1319. 10.1007/s00421-016-3384-0 27150353

[B17] Hanss R. Bein B. Ledowski T. Lehmkuhl M. Ohnesorge H. Scherkl W. (2005). Heart rate variability predicts severe hypotension after spinal anesthesia for elective cesarean delivery. J. Am. Soc. Anesthesiol. 102 (6), 1086–1093. 10.1097/00000542-200506000-00005 15915018

[B18] Ishaque S. Khan N. Krishnan S. (2021). Trends in heart-rate variability signal analysis. Front. Digital Health 3, 639444. 10.3389/fdgth.2021.639444 PMC8522021 34713110

[B39] Johnson A. (2009). Human performance: An ethnographic and historical account of exercise physiology. University of Pennsylvania.

[B19] Kenney W. L. Wilmore J. H. Costill D. L. (2022). Physiology of sport and exercise. Human kinetics.

[B20] Khan A. A. Lip G. Y. Shantsila A. (2019). Heart rate variability in atrial fibrillation: the balance between sympathetic and parasympathetic nervous system. Eur. J. Clin. investigation 49 (11), e13174. 10.1111/eci.13174 31560809

[B21] Kim K. K. Kim J. S. Lim Y. G. Park K. S. (2009). The effect of missing RR-interval data on heart rate variability analysis in the frequency domain. Physiol. Meas. 30 (10), 1039–1050. 10.1088/0967-3334/30/10/005 19713596

[B22] Li Z. Snieder H. Su S. Ding X. Thayer J. F. Treiber F. A. (2009). A longitudinal study in youth of heart rate variability at rest and in response to stress. Int. J. Psychophysiol. 73 (3), 212–217. 10.1016/j.ijpsycho.2009.03.002 19285108 PMC2719684

[B23] Lombardi F. (2002). Clinical implications of present physiological understanding of HRV components. Card. Electrophysiol. Rev. 6, 245–249. 10.1023/a:1016329008921 12114846

[B38] Liao P. He Q. Zhou X. Ma K. Wen J. Chen H. (2020). Repetitive bouts of exhaustive exercise induces a systemic inflammatory response and multi-organ damage in rats. Front. Physiol. 11, 685. 10.3389/fphys.2020.00685 32655413 PMC7324715

[B24] Maestroni L. Read P. Bishop C. Turner A. (2020). Strength and power training in rehabilitation: underpinning principles and practical strategies to return athletes to high performance. Sports Med. 50 (2), 239–252. 10.1007/s40279-019-01195-6 31559567

[B25] Makivić B. Nikić Djordjević M. Willis M. S. (2013). Heart Rate Variability (HRV) as a tool for diagnostic and monitoring performance in sport and physical activities. J. Exerc. Physiology Online 16 (3).

[B26] Maud P. J. Foster C. (2006). Physiological assessment of human fitness. Human kinetics.

[B27] Michael S. Graham K. S. Davis G. M. (2017). Cardiac autonomic responses during exercise and post-exercise recovery using heart rate variability and systolic time intervals—a review. Front. physiology 8, 301. 10.3389/fphys.2017.00301 PMC5447093 28611675

[B28] Moldovan I. Constantin A. Biagi P. Danila D. T. Moldovan A. Dolea P. (2015). The development of the Romanian VLF/LF monitoring system as part of the international network for frontier research on earthquake precursors (INFREP). Rom. Journ. Phys. 60 (7-8), 1203–1217.

[B29] Rahman S. Habel M. Contrada R. J. (2018). Poincaré plot indices as measures of sympathetic cardiac regulation: responses to psychological stress and associations with pre-ejection period. Int. J. Psychophysiol. 133, 79–90. 10.1016/j.ijpsycho.2018.08.005 30107195

[B30] Reganova E. Solovyeva K. Buyanov D. Gerasimenko A. Y. Repin D. (2023). Effects of intermittent hypoxia and electrical muscle stimulation on cognitive and physiological metrics. Bioengineering 10 (5), 536. 10.3390/bioengineering10050536 37237606 PMC10215293

[B31] Shaffer F. Ginsberg J. P. (2017). An overview of heart rate variability metrics and norms. Front. Public Health 5, 258. 10.3389/fpubh.2017.00258 29034226 PMC5624990

[B32] Tahara N. Takaki H. Taguchi A. Suyama K. Kurita T. Shimizu W. (2005). Pronounced HR variability after exercise in inferior ischemia: evidence that the cardioinhibitory vagal reflex is invoked by exercise-induced inferior ischemia. Am. J. Physiology-Heart Circulatory Physiology 288 (3), H1179–H1185. 10.1152/ajpheart.00045.2004 15498830

[B33] Thomas B. L. Claassen N. Becker P. Viljoen M. (2019). Validity of commonly used heart rate variability markers of autonomic nervous system function. Neuropsychobiology 78 (1), 14–26. 10.1159/000495519 30721903

[B34] Wehrwein E. A. Orer H. S. Barman S. M. (2016). Overview of the anatomy, physiology, and pharmacology of the autonomic nervous system. Compr. Physiol. 6, 1239–1278. 10.1002/cphy.c150037 27347892

[B35] Will P. M. Walter J. D. (1999). Exercise testing: improving performance with a ramped Bruce protocol. Am. heart J. 138 (6), 1033–1037. 10.1016/s0002-8703(99)70067-0 10577432

[B36] Wisneski L. (2017). A review of classic physiological systems. Sci. Basis Integr. Health 1-46, 1–46. 10.4324/9781315153889-2

[B37] Zhao S. Ait-Belaid K. Shen Y. Zhou K. (2024). The neurological effects of acute physical exhaustion on inhibitory function. Physiology and Behav. 284, 114641. 10.1016/j.physbeh.2024.114641 39019134

